# Possibilities and constraints of rapid online ethnography: Lessons from a rapid assessment of COVID-19 policy for people who use drugs

**DOI:** 10.3389/fsoc.2022.959642

**Published:** 2022-08-22

**Authors:** Emery R. Eaves, Robert T. Trotter, Bonnie Marquez, Kayla Negron, Eck Doerry, David Mensah, Kate A. Compton-Gore, Shana A. Lanzetta, Kathryn Kruithoff, Kaitlyn Dykman, Julie A. Baldwin

**Affiliations:** ^1^Department of Anthropology, Northern Arizona University, Flagstaff, AZ, United States; ^2^Center for Health Equity Research, Northern Arizona University, Flagstaff, AZ, United States; ^3^School of Informatics, Computing, and Cyber Systems, Northern Arizona University, Flagstaff, AZ, United States; ^4^Department of Health Sciences, Northern Arizona University, Flagstaff, AZ, United States

**Keywords:** COVID-19, substance use policy, medication for opioid use disorder (MOUD), rapid assessment response and evaluation (RARE), online ethnography

## Abstract

During the COVID-19 Pandemic, health care provision changed rapidly and funding became available to assess pandemic-related policy change. Research activities, however, were limited to contactless, online delivery. It was clear early on that some elements of online rapid ethnography were feasible and effective, while others would not approach traditional ethnographic depth. We conducted an online Rapid Assessment, Response, and Evaluation (RARE) project from August 2020 to September 2021 to understand how COVID-19 policy impacted people who use drugs. Our interdisciplinary research team conducted online ethnographic interviews and focus groups with 45 providers and community stakeholders, and 19 clients from rural and urban areas throughout Arizona. In addition, 26 webinars, online trainings, and virtual conferences focused on opioid policy and medication for opioid use disorders (MOUD) were opportunities to observe conversations among providers and program representatives about how best to implement policy changes, how to reach people in recovery, and what aspects of the changes should carry forward into better all-around opioid services in the future. Our RARE project was successful in collecting a range of providers' perspectives on both rural and urban implementation of take-home MOUDs as well as a wide view of national conversations, but client perspectives were limited to those who were not impacted by the policies and continued to attend in-person daily clinic visits. We describe challenges to online rapid ethnography and how online research may have allowed for an in-depth, but incomplete picture of how policy changes during COVID-19 policy affected people with opioid use disorders.

## Introduction

During the COVID-19 Pandemic, in response to providers and programs calling for more flexibility, the United States Drug Enforcement Administration (DEA) temporarily relaxed restrictions to serve patients in substance use disorder (SUD) treatment. Changes included longer take-home doses of methadone and buprenorphine, fewer barriers for prescriber authorization, and allowances for telehealth delivery (SAMHSA, 2020). These changes directly and indirectly impacted the approximately 14,500 substance use treatment programs in the United States, but the actual implementation of the changes varied.

In the months after the guidelines changed, media reports described enthusiasm among behavioral health providers regarding these policy changes and described the changes as what providers had been asking for (Eaves et al., 2020). Harm reduction programs argued that people who use drugs should have the medications they need and that telemedicine has potential not only to reduce the risk of COVID, but also to reduce burden on clients in general and address some of the difficulties and stigma associated with MOUDs, particularly methadone which typically requires daily dosing.

Initial research has suggested that access to take-homes and virtual visits decreased stigma, increased access to MOUD, and allowed providers the flexibility to engage in more patient-centered care (SAMHSA, 2021). Amidst these changes, healthcare providers and policymakers worked to manage crisis situations, particularly the competing public health emergencies represented by the opioid epidemic in the context of the COVID-19 pandemic (Pérez-Chiqués et al., 2021).

Housing insecurity, unstable employment, and related financial concerns are common among people who use drugs (Harris et al., 2019; Jemberie et al., 2020; Volkow, 2020). During the COVID-19 pandemic, closures and mandates increased social isolation, unemployment, and a range of stressors that elevated relapse risk for people in substance use recovery (Melamed et al., 2021). Individuals seeking substance use treatment, in many cases, reported encountering inactive phone lines, discontinued programs, or unresponsive clinics when seeking services, particularly in rural areas (Conway et al., 2022; Melamed et al., 2022). These issues are present in Arizona, and the demographic and geographic contexts made it an interesting case example to consider the impacts of SUD policy change.

Arizona has only two major metropolitan centers; thus, people living in rural areas face up to 5 hours driving distance to reach a medical specialist. Eighty percent of the population live in mental health professional shortage areas (Koppell et al., 2014), with few options for mental or behavioral healthcare in rural areas. Given this context, Arizona's telemedicine infrastructure was well-developed prior to the pandemic. Many clinics throughout the state were ready to immediately implement changes to allow patients more flexibility and access to SUD treatment through telemedicine (Rowe, 2020).

During the COVID-19 pandemic, access to online communication went from a luxury to a necessity, exacerbating the “digital divide” and further disadvantaging those with limited or no access to communication technology (Busch et al., 2021; Lai and Widmar, 2021; Cheshmehzangi et al., 2022). The “digital divide” is a term used to describe disparities between people who have access to communication technology and people who don't (Lythreatis et al., 2021). Particularly among people who are housing insecure or living in poverty, a large percentage of residents in inner city and rural areas don't have reliable internet access (Ramsetty and Adams, 2020; Reddick et al., 2020).

Due to circumstances also stemming from the COVID-19 pandemic, including university restrictions on research and clinic closures, our research on these changes was initially limited to virtual environments. Working in a mostly virtual domain revealed some surprising benefits as well as barriers to information access. Here, we describe complexities of both online care delivery for substance use disorders, as well as challenges inherent in online ethnographic research, and suggest key areas for future research.

## Methods

Rapid Assessment, Response and Evaluation (RARE) is a National Institutes of Health and National Centers for Disease Control and Prevention (NIH/CDC) sponsored/created methodological approach to providing institutions and communities information they need to respond to time sensitive crisis situations (Trotter et al., 2001; Needle et al., 2003; Trotter and Singer, 2005). RARE assessment involves triangulation of multiple methods to conduct rigorous, locally responsive assessment and evaluation within a much shorter timeframe than conventional research (Needle et al., 1999; Trotter and Singer, 2005, 2007; Bates et al., 2007). RARE methodology has been tested in various health crisis situations, including HIV prevention (Bates et al., 2007; Sabin et al., 2008), pandemic mitigation (Needle et al., 2003; Trotter and Singer, 2005), and substance use prevention and recovery (Stimson et al., 1999; O'Connell et al., 2005; Valderrama et al., 2006; Loosier et al., 2020).

Core RARE methods used in this project included community solicitation, expert interviews, focus groups, and participant observation (Trotter et al., 2001; Minkler and Wallerstein, 2011; Hardy et al., 2014). Recruitment for interviews and focus groups employed a standard qualitative sampling approach, which involves targeting individuals with expert knowledge or personal experience with substance use treatment during COVID-19 (Trotter, 2012). To engage a broad range of perspectives, in addition to reaching out to our community meeting participants for recommendations and referrals, our student researchers (KN, KD, DM) used google and AZ Department of Health listings to compile contact information for 54 opioid treatment centers from all 15 counties in the state. The students reached out to each by email and by phone, and asked respondents to refer their interested colleagues and clients. We placed a link and flier for our study on some of the larger clinic groups in the state, and met with directors of large multi-clinic agencies to discuss strategies for reaching their clients and providers. Participants included people in substance use treatment (clients), providers, payers, and leaders of stakeholder organizations. Our questions were designed around RARE domains to assess: (1) risk and protective factors; (2) contextual factors (environment); and (3) currently available programs and how to improve them (Trotter et al., 2001). Assessment of contextual factors at individual, interpersonal, community, and policy levels were guided by social ecological understandings (Bronfenbrenner, 1994).

Our interpretation is based on interviews and focus groups with 19 clients and 45 providers in Arizona. Some focus groups were conducted with groups of providers or clients within a single organization. For example, one group invited us to conduct a focus group as part of their weekly provider meeting. Others were pre-scheduled and advertised so anyone interested could respond and receive a link to participate online. Interviews and focus groups were conducted by phone or zoom due to COVID-related restrictions. Our research team also attended trainings, webinars, information sessions, and other conversations between providers, policy-makers, and researchers that were available throughout the project to gain a broader perspective on national and state-level approaches to policy implementation. The authors undertook 26 episodes of participant observation at events and recorded findings in field notes which also informed our analysis.

As a community-engaged approach, a first step in conducting RARE is to understand local interest, priorities, and questions. We convened community stakeholders (payers, local leaders, providers, policy-makers) to discuss perceptions of risks, needs, and impacts of COVID-19 policy changes. Qualitative interviews with providers, program directors, and clients, and other stakeholders were an opportunity to delve deeper into issues encountered in implementation of the new guidelines and individual and interpersonal level barriers and facilitators to telehealth and mHealth care delivery. RARE focus groups were an additional and more conversational way to learn what challenges people encountered with implementation of the new guidelines; what supports they found most useful in implementing the new guidelines; and what policies and procedures they have implemented to evaluate safety and suitability of take-home MAT doses for patients.

Our team included medical anthropologists, public health researchers, counselors, computer scientists, and graduate and undergraduate students. Interview and focus group recordings were transcribed verbatim and analyzed using ATLAS.ti qualitative data analysis software. A team of 7 coders (EE, KN, KD, KK, DM, BM, KCG) developed a codebook based on reading through and discussing transcripts. The coding team met regularly after individually coding the same transcript to discuss each code. We conducted 4 rounds of this process until consensus was reached on the use of each code, final codes were determined, and coders reached consensus about the use and application of each code. After coding all transcripts in ATLAS.ti, 2 coders went back over all transcripts to check that codes were consistent (KN and DM). Next, the team generated code reports and summarized and discussed results to identify initial emergent themes. The team created a coding memo, or description of key themes in each code report, for each code.

All research procedures were approved by the Northern Arizona University Human Subjects Review Board and all participants provided informed consent. When attending webinars or online meetings, we explained our research during initial introductions if conversations were part of the process. In public webinars that did not include audience participation, we did not announce our presence. We did not treat the notes from these meetings as data. Attendance was to inform our broader understanding of the context. These observations were included in our ethical approval.

## Results

It was clear early in our research that some elements of online RARE were feasible and effective, while others were difficult to approximate. Online conferences, webinars, and trainings, for example, offered an opportunity for our team to gain local and national perspectives on implementation, and to participate in conversations without expensive and time-consuming travel. We were able to convene providers and program directors from throughout the state in a single community meeting, only requiring an hour of their time, a clear advantage of online ethnography. Providers and program representatives engaged in meetings and interviews and enthusiastically shared their perspectives on the guidelines and the national conversation about policy change as it took shape. Clients or people in recovery, on the other hand, were protected by the US Health Insurance Portability and Accountability Act (HIPAA) privacy protections against information sharing, email policies, telemedicine appointments, and were subsequently difficult to reach.

RARE methods have been instrumental in informing drug policy and gathering in-depth data over short periods. RARE projects have involved street intercept surveys, where a research assistant stands on a street corner or area where people who use drugs are present and asks them to respond to survey questions as they have time (Needle et al., 1999, 2000). RARE has also successfully employed and trained local people as researchers to conduct ethnographic research from an insider's perspective, more quickly gaining the trust and collaboration of the community (Trotter et al., 2001; Trotter and Singer, 2005; Hardy et al., 2015). We tried several ways to mimic the process of a street intercept survey. We posted a qualtrics survey on social media, linked on the sites of some of the most well-known harm reduction and clinical organizations in the state, but quickly found once again that the possibility of internet scams was a major barrier, getting scammed ourselves in the form of 4500 fake survey responses completed within an 8 h period.

Despite our efforts to bridge the digital divide, our results do not include the voices of individuals who were not in treatment, who were unable to access telemedicine, or who encountered closed clinics or dead phone lines. Like many clinics during the pandemic shutdown, we could only engage online for the majority of our project, which started in August 2020 and lasted until summer 2021. This online-only presence limited our reach to clients who came in-person to clinics throughout the pandemic, or who successfully engaged in online or telehealth-based treatment. Emergent themes in our data show important aspects of telemedicine and clinic experiences in the context of COVID-19, as well as highlight how the digital divide emerged as a key barrier to online ethnographic research and to telemedicine as a primary method of treatment.

### Many things in today's world have shifted to an online platform. Why not ethnography too?

Traditionally, participant observation is conducted in person in “the field.” Some aspects of context can be observed only in person rather than in a virtual environment. On the other hand, virtual environments offer a range of advantages that many researchers capitalized on during the COVID-19 pandemic. For example, online platforms allowed us to reach people around the state without the need for extensive travel. The transition from in-person to online formats for trainings, meetings, seminars, and other interactions also offered a broader range of participant observation opportunities than we would have encountered in traditional ethnography. These meetings included virtual webinars, conferences, trainings, and town hall style meetings. In contrast to ethnographic interviews, where participants are responding to direct questions, or focus groups where participants are discussing a research-posed question, webinars, conferences, and trainings were participant-organized and became a key platform to understand how providers were engaging with one another, views on the changes, and how providers were training one another to navigate treatment contexts during a pandemic.

#### Providers' perspectives

Providers described benefits and drawbacks to meeting with clients online during the pandemic. Online consultations reduced the need for long-distance travel, allowing some providers to reach more patients. Several providers described frustration, however, with not being able to induct new patients without in-person consultations.

*I would like to see the policy be reconsidered you know, maybe they do some sort of thing where if the client is over 50 miles from you they can forego that initial face-to-face appointment and be prescribed Suboxone initially via telemedicine. I don't know. I think that is definitely something to be looked at to see how we can get some of these clients earlier access to care without an actual face-to-face appointment. (Provider)*

Although providers described many positive outcomes of meeting clients *via* telemedicine or virtual platforms, without face-to-face interaction, even for existing clients, providers described a lack of personal connection and difficulty reading the postures, body language, and overall wellbeing of clients. One provider explained it this way:

*Effective behavioral health care relies a lot on nonverbal behaviors and cues. Relies on smell frankly. I mean not necessarily in a bad way just you can tell a lot about what's coming in your nose, you can tell a lot by hesitancy or lack of hesitancy. You can tell a lot by the way somebody sits in a chair, I mean and when they're in your office you can assume they're undistracted. But we know, people are sitting in their cars, there you know sitting on park benches are sitting in living rooms, with their significant other, on the other side of the room, I mean it's a very different kind of experience*.

Providers also struggled to assess their clients' overall health *via* telemedicine, noting often that drug screening was a key challenge during the pandemic because it could not be done online. Providers cited HIPAA and privacy protections as important, but imposing barriers on their ability to reach patients because it was difficult to share information or coordinate care. The description below illustrates multiple issues noted by many providers, including the difficulty of obtaining accurate drug screens, limited monitoring, and inability to coordinate care through privacy protections.

*[One of my clients] has been doing telemedicine since June and we just found out he's actually been using fentanyl the whole time and selling his Suboxone. And nobody knew because we weren't having eyes on him. He wasn't coming to therapy; he had actually gotten out of a rehab facility and was doing out-patient services there and not our agency and we couldn't release of information to coordinate care with the other agency to see if he was still attending*.

#### Research team's perspectives

Reaching providers online was not easy. Phone calls and emails were primary modes of communication available to our team. Emailing stakeholders and clinic directors about our research project yielded the most responses. Phone calls would often go to voicemail, or a person at the front desk would take a message, promising to pass it on. These often produced no results and despite following up, we would not hear back.

One of the key challenges in virtual ethnographic outreach and communication was a concurrent increase in email communication generally, as well as the increasing fraudulent or soliciting emails providers receive, leading our communication to be easily dismissed as fake or simply forgotten. To address these shortcomings in online communication, once restrictions on in-person interaction shifted, a graduate research assistant member our team (DM) attempted in-person outreach to try reaching clinic directors and managers face-to-face. What he encountered were many closed doors due to the COVID-19 pandemic, as many clinics had shifted entirely to a virtual environment. Those clinics that were open were often reluctant to pass on messages or to allow the researcher to hand out surveys or connect with their already overburdened providers.

Clients involved in MOUD treatment were difficult to recruit in an online environment. This population experiences health disparities that have led them to distrust the virtual world and there are many protections set up for their privacy. Clients responded most often to flyers that were posted at local clinics they visited in person. This meant that clients who continued to visit in-person, open clinics were the people who encountered our recruitment materials. Providers told us in many cases that although they thought their clients would be interested in sharing their experiences, they were not allowed to email clients, which made it difficult to communicate about our study unless they were meeting in person.

#### Clients' perspectives

Clients we reached who were involved in telemedicine treatment and take-home doses of MOUD through provider networks expressed satisfaction with the impacts of the changes and additional take-home allowances.

*Yeah, I don't get to see them in person, but it hasn't really affected me because I still get the same end goal out of it. I'm able to discuss my dosage, discuss what's working for me and what's not, and what my goal is, and there's the same outcome. The only thing that changed was being face to face, but I don't feel like it made my experience any less*.

Other clients described frustration with technology difficulties, lack of personal connection, or other minor issues. Clients also expressed frustration with the limitations of required drug screening, which they had to complete in person even to engage in online treatment appointments. Clients noted many drawbacks and difficulties with the continued need for in-person urine analysis. Reductions in clinic staff was a factor that clients felt negatively impacted privacy and security in the screening process, and as providers explained as well, many clients felt the in-person drug screens were limiting the benefits they received from engaging in telemedical care to avoid COVID-19 contagion.

Many clinics adapted to parking lot dosing or other creative ways to have clients come in without risking COVID-19 exposure. Clients described many positive interactions with providers during pandemic closures, noting that creative measures often enhanced their sense of being cared for and being able to access support networks. Others described being required to attend in-person clinic visits throughout closures, even while their providers were not in person and clients sat in the clinic talking to a provider over Zoom. One client said, for example, “Telemedicine has been used to allow staff and doctors to stay home and avoid risk while clients still have to come in to the empty room to speak to a computer.”

### What's missing in online ethnography?

Online substance use treatment resources offered a way to reduce contagion, alleviate the burden of travel for people in rural areas, and increase continuity of care for clients who moved away. The provider quoted below noted difficulty and access issues, but argued that Arizona clinics have been making great strides toward addressing some of these. She stated that continued flexibility could be a step toward addressing digital and transportation inequity.

*Because the access to care issues that COVID brought about were already an issue in rural populations and the social determinants of health and people who are who are too poor to afford fancy technology, or people who don't have a way to transport themselves into the clinic. And, of course, the Internet and electronic access too. Those were already there and already a problem and then COVID of course made that so much worse. And so we found all these really great solutions which are really working and to lose them and lose what little we gained would be just devastating and access in Arizona has been just wonderful about supporting the long term use of some of these innovations*.

What was missing in both online treatment, and in online ethnographic assessment, however, was access to clients that were not online. Bridging gaps in transportation through virtual interaction is promising, but also requires investment in addressing the widening gap between people with access to digital technology and people without.

## Conclusion

Understanding more about implementation of MOUD-related guideline changes and equity in access to “take-homes” for people in rural and underserved populations was a primary focus of our project. To evaluate attitudes toward and implementation of the new guidelines in a variety of programmatic contexts, we designed our study to investigate institutional procedures and provider attitudes toward MAT prescribing changes in relation to a post-COVID-19 environment.

Online RARE methods were a useful way to gain insight into the experiences of people who transitioned to online services in the context of a global pandemic. At the same time, online ethnography is limited to engagement on only one side of the digital divide. People seeking novel treatment (not existing clients) may have encountered closed doors at clinics that were offering treatment in an entirely virtual format during pandemic lockdown. We found that telemedicine offered a promising way to address transportation barriers and connect people in spite of closures and distance. On the other hand, it was difficult to recruit a broad range of clients and providers online, and our reach was limited to people already engaged in these services. In a post-pandemic context, online ethnographic methods may be better combined with in-person methods, or limited to understanding those engaging in online environments.

Additional research is needed to understand the experiences of those who sought treatment during the pandemic and encountered closed doors, insurmountable technological barriers, or empty group support chairs. As digital ethnography gains popularity alongside big data analysis and reliance on medical records of those engaged in the system, those not engaging in online platforms and existing health systems may be left out. Our findings suggest that innovative ways to protect privacy without isolating people are needed as protection also serves to disconnect people who use drugs not only from treatment services they may want, but also from sharing their stories and voices to contribute to policy improvement more broadly.

## Data availability statement

The datasets presented in this article are not readily available because data are from qualitative interviews cannot be sufficiently anonymized to share in full. Requests to access the datasets should be directed to emery.eaves@nau.edu.

## Ethics statement

The studies involving human participants were reviewed and approved by Northern Arizona University Institutional Review Board. The Ethics Committee waived the requirement of written informed consent for participation.

## Author contributions

EE designed the study, oversaw data collection and interpretation, and finalized the manuscript. RT helped design the study and provided methodological expertise to the interpretation of results, and approved the final manuscript. BM managed recruitment and was involved in data collection, coding, analysis and helped draft the initial manuscript. KN was involved in data collection, coding, and analysis and helped draft the initial manuscript. ED was involved in study design and provided methodological guidance and expertise and approved the final manuscript. DM, KC-G, KK, and KD were involved in data collection, coding, and analysis and approved the final manuscript. SL was involved in recruitment, data collection and analysis, and approved the final manuscript. JB was the principal investigator for the project and helped with study design and approved the final manuscript. All authors contributed to the article and approved the submitted version.

## Funding

This work was supported by the National Institutes of Health, National Institute for Minority Health and Health Disparities as a supplement to the Southwest Health Equity Research Collaborative NIH/NIMHD RCMI U54MD012388-04S4 (JB-PI).

## Conflict of interest

The authors declare that the research was conducted in the absence of any commercial or financial relationships that could be construed as a potential conflict of interest.

## Publisher's note

All claims expressed in this article are solely those of the authors and do not necessarily represent those of their affiliated organizations, or those of the publisher, the editors and the reviewers. Any product that may be evaluated in this article, or claim that may be made by its manufacturer, is not guaranteed or endorsed by the publisher.
